# Trends in Linezolid Prescription in Japan based on National database open Data: implications for the need for therapeutic drug monitoring

**DOI:** 10.1186/s40780-026-00552-8

**Published:** 2026-02-11

**Authors:** Yuichi Muraki, Norio Ohmagari

**Affiliations:** 1 Laboratory of Clinical Pharmacoepidemiology, Kyoto Pharmaceutical University, Kyoto, 607-8414 Japan; 2 AMR Clinical Reference Center, National Center for Global Health and Medicine Japan Institute for Health Security, Tokyo, 162-8655 Japan

**Keywords:** Linezolid, Therapeutic drug monitoring, Prescription trends, National database open Data, Thrombocytopenia, Drug safety

## Abstract

**Background:**

Linezolid is an oxazolidinone antibiotic widely used to treat infections caused by resistant Gram-positive bacteria. It has long been regarded as a drug that does not require therapeutic drug monitoring, owing to its limited pharmacokinetic variability. However, recent clinical observations suggest that delayed linezolid elimination is associated with hematological toxicity and thrombocytopenia, particularly in older adults and individuals with reduced renal function. As Japan has a rapidly aging population, understanding real-world linezolid prescription patterns is important for contextualizing potential safety concerns related to its use.

**Methods:**

This study analyzed Japanese national linezolid prescription trends using publicly available data from the National Database of Health Insurance Claims. The annual prescription counts of oral and parenteral linezolid formulations were descriptively examined in inpatient and outpatient settings, along with age- and sex-specific distributions. To provide comparative context, inpatient trends in parenteral use of major anti- methicillin-resistant *Staphylococcus aureus* (MRSA) agents were also assessed using the same data source.

**Results:**

Inpatient use declined steadily from 2014 to 2023. Outpatient use remained extremely limited for most of the study period; however, oral outpatient prescriptions became observable from 2022 onward, though the absolute magnitude remained small. Given that outpatient care generally involves less intensive clinical monitoring, even limited outpatient use warrants careful interpretation. Age-stratified analysis revealed that prescriptions were concentrated among older adults, with the highest use observed in individuals aged 85–89 years. These patients are more likely to have reduced renal function, which can further increase drug exposure and the risk of toxicity. Stratified analyses further demonstrated that outpatient oral use was almost exclusively observed among older adults. In the comparative inpatient analysis of parenteral anti-MRSA agents, trends differed by agent, indicating that the decline in linezolid use did not simply reflect uniform changes in inpatient anti-MRSA antibiotic utilization.

**Conclusions:**

These national prescribing patterns indicate that linezolid continues to be used predominantly among older adults. Although causal relationships cannot be inferred from this descriptive analysis, the findings highlight clinical contexts in which careful monitoring of linezolid therapy may be particularly important. Further evaluation is warranted to determine whether monitoring strategies, including the potential role of therapeutic drug monitoring, could contribute to safer use of linezolid in high-risk patient populations.

## Background

Linezolid is an oxazolidinone antibiotic widely used to treat infections caused by methicillin-resistant *Staphylococcus aureus* (MRSA) and vancomycin-resistant *Enterococcus* spp. [1]. Historically, linezolid has been regarded as not requiring therapeutic drug monitoring (TDM), owing to its predictable pharmacokinetics and relatively wide therapeutic window [2]. However, accumulating evidence indicates that excessive trough concentrations are associated with hematologic toxicity, and particularly with thrombocytopenia [3–5]. Impaired renal function has also been identified as a major risk factor due to reduced nonrenal clearance pathways [3, 5].

In Japan, the aging population and the high prevalence of chronic kidney disease have raised concerns that real-world linezolid use may involve substantial numbers of high-risk patients [6]. Despite these concerns, national-level data describing the characteristics of linezolid users remain limited. The National Database of Health Insurance Claims and Specific Health Checkups of Japan (NDB) Open Data provides comprehensive information on prescription patterns across Japan and is suitable for evaluating real-world antimicrobial use [7, 8].

This study aimed to describe trends in linezolid prescriptions in Japan using NDB Open Data and to assess whether its usage patterns suggest clinical contexts in which additional monitoring strategies may warrant consideration.

## Main text

### Methods

Prescription data for linezolid were obtained from the NDB Open Data database, which provides nationwide aggregated statistics on drug prescriptions by formulation, care setting, age group, and sex [9]. The study period covered fiscal years 2014 through 2023. Annual prescription counts of oral and parenteral linezolid formulations were extracted, and age- and sex-stratified data were used to characterize demographic prescribing patterns.

Linezolid use was quantified using the defined daily doses per 1000 inhabitants per day (DDDs/1,000 inhabitants/day; DID), in accordance with the methods recommended by the World Health Organization [7]. Population data used for rate calculations were obtained from the Population Estimates published by the Statistics Bureau of Japan [10]. To provide minimal comparative context within the same analytic framework and data source, inpatient trends in parenteral use of major anti-MRSA agents were additionally evaluated. Annual prescription data for parenteral vancomycin, teicoplanin, daptomycin, arbekacin, tedizolid, and linezolid were extracted from the NDB Open Data for fiscal years 2014–2023. Consumption was converted to DID (DDDs/1,000 inhabitants/day) using WHO-defined DDDs and population estimates, consistent with the approach used for linezolid.

All analyses were descriptive in nature, summarizing trends in inpatient and outpatient prescriptions as well as demographic distributions. As the NDB Open Data are fully anonymized and lack individual-level clinical information, no adjustments for patient characteristics or inferential statistical analyses were performed. Ethical approval was not required because only publicly available aggregated datasets were analyzed.

## Results

In total, 1,438,625 oral and 2,461,862 parenteral linezolid prescription units were identified between the fiscal years 2014 and 2023. Of these, inpatient prescriptions accounted for 1,303,666 (90.6%) oral and 2,459,065 (99.9%) parenteral units, whereas outpatient prescriptions accounted for 134,959 (9.4%) oral and 2797 (0.1%) parenteral units.

**Fig. 1:**
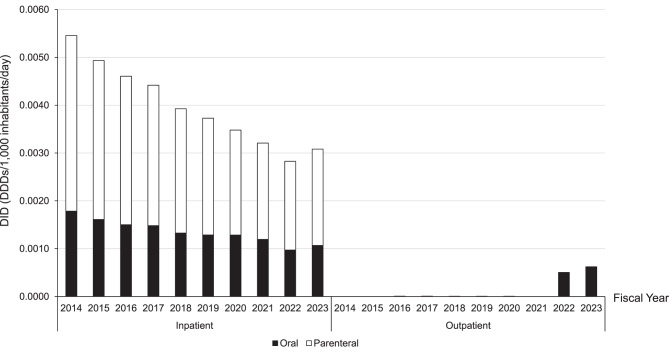
Trends in the use of linezolid from Fiscal Year 2014 to Fiscal Year 2023. Values of zero in outpatient settings indicate that prescription volumes are below the disclosure threshold of the NDB Open data and do not necessarily represent the complete absence of use

For inpatients, the total DID decreased from 0.0055 in 2014 to 0.0031 in 2023. A similar decreasing trend was observed for both the oral and parenteral formulations, with no notable differences between the dosage forms (Fig. 1). Outpatient prescriptions remained limited throughout most of the study period; however, oral formulations began to appear in the outpatient setting from 2022 onward. Outpatient DID values remained extremely low (≤0.00062), and outpatient parenteral use was nearly negligible across all fiscal years.

**Fig. 2:**
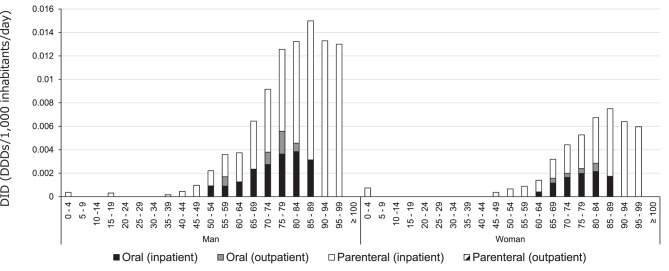
Linezolid usage by age group in fiscal Year 2023. Values of zero in outpatient settings indicate that prescription volumes are below the disclosure threshold of the NDB Open data and do not necessarily represent the complete absence of use

Age- and sex-stratified analyses showed that linezolid was prescribed predominantly to older adults (Fig. 2). Stratified analyses by care setting and formulation further revealed that outpatient oral linezolid use was almost exclusively observed among older adults, whereas outpatient parenteral use remained minimal across all age groups. In both men and women, prescription rates peaked in the 85–89 year age group. The proportion of prescriptions among individuals aged 65 years or older was 88.5%. The use of parenteral formulations increased with age.

**Fig. 3:**
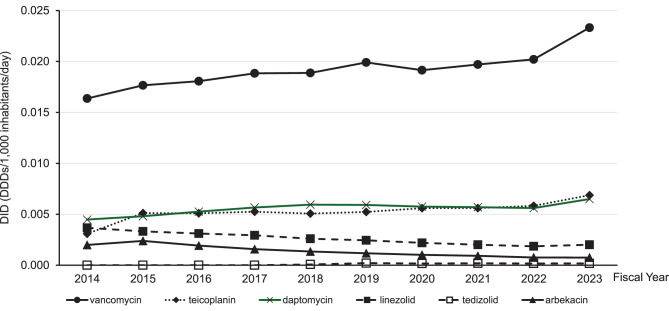
Trends in inpatient parenteral use of anti-MRSA agents from fiscal year 2014 to fiscal year 2023

In the comparative analysis of inpatient parenteral anti-MRSA agents (Fig. 3), vancomycin showed the highest DID throughout the study period and demonstrated a gradual increase. Daptomycin and teicoplanin exhibited relatively stable trends with slight increases, whereas linezolid showed a gradual decline. Arbekacin and tedizolid remained at substantially lower levels throughout the study period.

## Discussion

This descriptive epidemiological analysis using the NDB Open Data clarifies national trends in linezolid use in Japan over a 10-year period. Although inpatient DID values declined during much of the study period, multiple factors may have contributed to this decline, including the availability of tedizolid as an alternative oxazolidinone agent [11], the nationwide promotion of antimicrobial stewardship [12], declining MRSA isolation rates [13], reductions in the number of patients requiring anti-MRSA therapy [14], and changes in healthcare utilization patterns during the COVID-19 pandemic [15]. Although detailed analyses of tedizolid prescribing patterns were not the primary focus of the present study, its availability as an alternative oxazolidinone agent may have influenced linezolid prescribing patterns. Despite these influences, linezolid continued to be prescribed consistently until 2023, indicating that it remains an important therapeutic option in clinical practice, particularly for infections caused by resistant Gram-positive pathogens.

Importantly, the comparative inpatient analysis using the same NDB Open Data demonstrated heterogeneous trends across major anti-MRSA agents (Fig. 3). While inpatient parenteral use of vancomycin, daptomycin, and teicoplanin was stable or increased over time, linezolid showed a gradual decline. This within-dataset comparison indicates that the observed decline in linezolid use is unlikely to be explained solely by overall shifts in inpatient anti-MRSA antibiotic utilization and instead suggests agent-specific prescribing dynamics. These findings are consistent with prior reports suggesting agent-level heterogeneity in anti-MRSA agent use in Japan [16, 17].

The age distribution revealed that 88.5% of all prescriptions occurred in individuals aged 65 years or older, with the highest use observed among those aged 85–89 years. Given that renal function declines with age [18] and that impaired clearance of linezolid has been associated with elevated drug exposure and hematologic toxicity [3–5], these findings indicate that a substantial proportion of real-world prescriptions involves patients at increased risk of adverse effects. Stratified analyses further revealed that outpatient oral linezolid use was concentrated almost entirely among older adults. Although it remains unclear whether this age distribution is unique to linezolid or shared by other anti-MRSA agents, this pattern is clinically relevant. In particular, linezolid has traditionally been administered as a fixed-dose agent without routine therapeutic drug monitoring, and its predominant use among older outpatients—who may have limited opportunities for laboratory monitoring—raises important drug safety considerations.

The emergence of outpatient oral prescriptions beginning in 2022 is also noteworthy. However, interpretation of outpatient trends requires caution. In the NDB Open Data, drugs with prescription volumes below predefined thresholds are not disclosed, and the number of listed products varies by fiscal year (top 30 products in 2014, top 100 products from 2015 to 2021, and top 100–500 products depending on therapeutic category from 2022 onward) [9]. Consequently, outpatient linezolid use in earlier years may have been underestimated or unobserved due to data suppression rather than a true absence of prescribing. Even so, outpatient oral use, though limited in magnitude, may be clinically meaningful given the restricted opportunities for laboratory monitoring in outpatient settings [19].

Several limitations should be noted. The NDB Open Data does not include clinical diagnoses, dosing information and treatment outcomes. In addition, prescriptions data may be underestimated because products with very low prescription volumes are not disclosed to ensure patient anonymization. Despite these limitations, this study provides the first nationwide description of long-term linezolid prescribing trends in Japan and offers a basis for future studies evaluating safety monitoring strategies.

## Conclusions

This study describes national trends in linezolid use in Japan and showed that the drug continues to be prescribed predominantly among older adults. Although the present analysis does not establish a clinical necessity for TDM, the findings suggest that further evaluation of monitoring strategies, including the potential role of TDM, may be warranted to support the safe and effective use of linezolid in high-risk patient populations.

## Data Availability

The datasets used and/or analyzed in the current study are available from the corresponding author upon reasonable request.

## Abbreviations

MRSA: Methicillin-resistant *Staphylococcus aureus*
TDM: therapeutic drug monitoring
NDB: National Database of Health Insurance Claims and Specific Health Checkups of Japan
DID: defined daily doses per 1000 inhabitants per day
